# Current Status of Human Papillomavirus Infection and Cervical Cancer in the Philippines

**DOI:** 10.3389/fmed.2022.929062

**Published:** 2022-06-20

**Authors:** Ryan C. V. Lintao, Leslie Faye T. Cando, Glenmarie Angelica S. Perias, Ourlad Alzeus G. Tantengco, Ian Kim B. Tabios, Clarissa L. Velayo, Sheriah Laine M. de Paz-Silava

**Affiliations:** ^1^Multi-Omics Research Program for Health, College of Medicine, University of the Philippines Manila, Manila, Philippines; ^2^Institute of Biology, College of Science, University of the Philippines Diliman, Quezon City, Philippines; ^3^Institute of Human Genetics, National Institutes of Health, University of the Philippines Manila, Manila, Philippines; ^4^Department of Physiology, College of Medicine, University of the Philippines Manila, Manila, Philippines; ^5^Division of Maternal-Fetal Medicine, Department of Obstetrics and Gynecology, College of Medicine and Philippine General Hospital, University of the Philippines Manila, Manila, Philippines; ^6^Department of Medical Microbiology, College of Public Health, University of the Philippines Manila, Manila, Philippines

**Keywords:** human papillomavirus, cervical cancer, epidemiology, vaccination, screening, treatment, Philippines

## Abstract

Cervical cancer is estimated to cause 341,831 deaths each year, with 9 of 10 deaths occurring in developing countries. Over the past decade, there has been a significant increase in cervical cancer incidence among women in the Philippines. Persistent infection with high-risk human papillomavirus (HPV) is the well-established necessary cause of cervical cancer. Based on limited studies conducted in the Philippines, the prevalence of infection with any HPV genotype was 93.8% for cervical squamous cell carcinoma and 90.9% for cervical adenocarcinomas. HPV types 16 and 18 were the most common HPV genotypes among Filipino patients with cervical cancer. On the other hand, the incidence of HPV infection among Filipino women with normal cervices was 9.2%. The World Health Organization has launched a global agenda of eliminating HPV infection by 2030. One of its key milestones is to vaccinate 90% of girls with the HPV vaccine by 15 years. However, the HPV vaccination rate among Filipino women remains to be unsatisfactory. HPV vaccination has only been included in the Philippine Department of Health's community-based National Immunization Program in 2015. Despite these efforts, the Philippines currently ranks last on HPV program coverage among low-middle income countries, with coverage of only 23% of the target female population for the first dose and 5% for the final dose. The principal reason for the non-acceptance of HPV vaccines was the perceived high cost of vaccination. The low utilization of available cervical cancer screening tests such as Pap smear and visual inspection with acetic acid hampered the Philippines' control and prevention of HPV infection and cervical cancer. Among those diagnosed with cervical cancer in the Philippines, only an estimated 50% to 60% receive some form of treatment. To this end, we summarize the burden of HPV infection and cervical cancer on Filipinos and the risk factors associated with the disease. We present the current screening, diagnostics, treatment, and prevention of HPV-related diseases in the Philippines. Lastly, we also propose solutions on how each building block in health systems can be improved to eliminate HPV infection and reduce the burden of cervical cancer in the Philippines.

## Introduction

Human papillomavirus (HPV) infection is the most common sexually transmitted infection worldwide. Aside from causing anogenital warts, persistent infection with high-risk HPV genotypes is the well-established necessary cause of cervical cancer (1). It is estimated that 604,127 new cases of cervical cancer occur each year, 88% of which occur in low- and middle-income countries. Cervical cancer is also estimated to cause 341,831 deaths each year; 9 of 10 deaths occur in developing countries (2).

Currently, cervical cancer is the fourth leading cancer in women worldwide and the second most common cancer among women of reproductive age (2). Cervical cancer is a preventable disease, owing mainly to HPV vaccines, screening, and treatment for early premalignant lesions. Three HPV vaccines have been approved for clinical use—a quadrivalent vaccine derived from HPV types 6, 11, 16, and 18; a bivalent vaccine derived from HPV types 16 and 18; and a nonavalent vaccine that provides additional coverage to HPV types 31, 33, 45, 52, 58—to prevent cervical, vulvar, and vaginal cancers and their precancerous lesions. All three vaccines have been effective in decreasing infection rates of high-risk vaccine genotypes (3–5).

The World Health Organization (WHO) has launched a global agenda of eliminating HPV by 2030. One of its key milestones is to vaccinate 90% of girls with HPV vaccines by 15 years of age (6). Despite the availability of HPV vaccines since 2006, only 107 (55%) of the 194 WHO member states have introduced HPV vaccine in their national immunization program. The Americas and Europe are the regions with the most introductions (85 and 75%, respectively), whereas Asia and Africa have the least (40 and 31%, respectively) (7).

In the Philippines, 37.8 million women are at risk for cervical cancer. The country has an annual burden of 7,897 cervical cancer cases and 4,052 deaths (8). However, HPV vaccination has only been included in the Department of Health's community-based National Immunization Program in 2015 (9). This was followed by a school-based HPV vaccination program in pilot elementary schools launched in 2017 to reach school-age girls (10). Despite these efforts, the Philippines currently ranks last on HPV program coverage among low-middle income countries (7), with coverage of only 23% of the target female population for the first dose and 5% for the final dose (11). Thus, there is much to do to achieve the goal of HPV elimination. There is a need to strengthen monitoring of HPV infection and disease, assess the progress of HPV vaccination programs, and assess the impact of current treatment practices on the Filipino population. To this end, we summarize the existing data on the burden of HPV and cervical cancer on Filipinos and the risk factors associated with the disease. We present the current screening, diagnostics, treatment, and prevention of HPV-related diseases in the Philippines. Lastly, we also propose solutions on how each building block in health systems can be improved to meet the global HPV agenda.

## HPV: The Necessary Cause

The causal link of HPV infection with cervical cancer has long been established (12). HPV has a global prevalence of 11% in women without cervical abnormalities and 99.7% in women with cervical carcinomas (1, 13). There are numerous genotypes of HPV, causing anogenital and non-genital warts, but carcinogenesis is mostly seen in high-risk or carcinogenic genotypes. Specifically, HPV types 16 and 18 are responsible for at least 70% of cervical cancer cases worldwide, while HPV types 31, 33, 35, 45, 52 and 58 contribute around 20% of the cases (1, 11, 14). In the Philippines, a pioneer case-control study showed that HPV DNA was detected in 93.8% of squamous cell cervical carcinoma cases and 90.9% of cervical adenocarcinoma cases compared with 9.2% of controls. HPV type 16 was the most common genotype found in patients with cervical cancer, followed by HPV types 18 and 45 (15). HPV types 16 and 18 have a prevalence of 21.2% among those with low-grade squamous intraepithelial lesions (LSIL) determined *via* cervical cytology, 42.1% among those with high-grade squamous intraepithelial lesions (HSIL), and 58.6% among women with cervical cancer (7, 15). A more recent case-control study showed that 75% of cervical cancer patients were positive for HPV types 16, 18 or 52. On the other hand, 25% of patients with non-malignant cervices were also positive for HPV types 16, 18 or 52. HPV types 18 and 52 were only detected in cervical cancer patients and not in control (16).

Persistent infection by high-risk genotypes has been implicated in malignant transformation in cervical cancer, facilitated by viral oncoproteins E6 and E7 (17–19). Both proteins function in shifting the infected cell to a proliferative state needed to support viral replication. HPV E6 induces degradation of p53 *via* the ubiquitin-proteasome pathway, resulting in inhibition of apoptosis in the infected cell despite eventual accumulation of genetic mutations (17, 20). HPV E7, on the other hand, is associated with retinoblastoma protein (pRB) (21, 22). In a normal cell, pRB acts as a repressor of E2F, a critical factor in cell cycle progression from G1- to S-phase. Association of HPV E7 to pRB releases E2F, which acts as a transcription factor to activate cellular entry to S-phase (23, 24). Furthermore, the integration of viral genes into the host chromosome further contributes to continuous E6 and E7 expression, subsequent genomic instability, and accumulation of mutations (25).

While the mechanism behind progression of cervical intraepithelial neoplasia (CIN) 1 to CIN 3 remains unclear, it is hypothesized that increased expression of E6 and E7 by high-risk HPV genotypes influences cellular progression to carcinogenic phenotypes. This contrasts with the low E6 and E7 expression levels in CIN 1 lesions (13). Accumulated genomic disturbances in high-grade lesions also allow the integration of viral genomes into the chromosome of host cell. This leads to further destabilization of chromosomal areas and increased expression of HPV oncogenes (26, 27). Cells with integrated viral genomes lose multiple gene regulatory mechanisms, and clones are later selected for neoplastic growth (28, 29).

## Risk Factors for HPV Infection and Cervical Cancer in the Philippines

**Figure 1:**
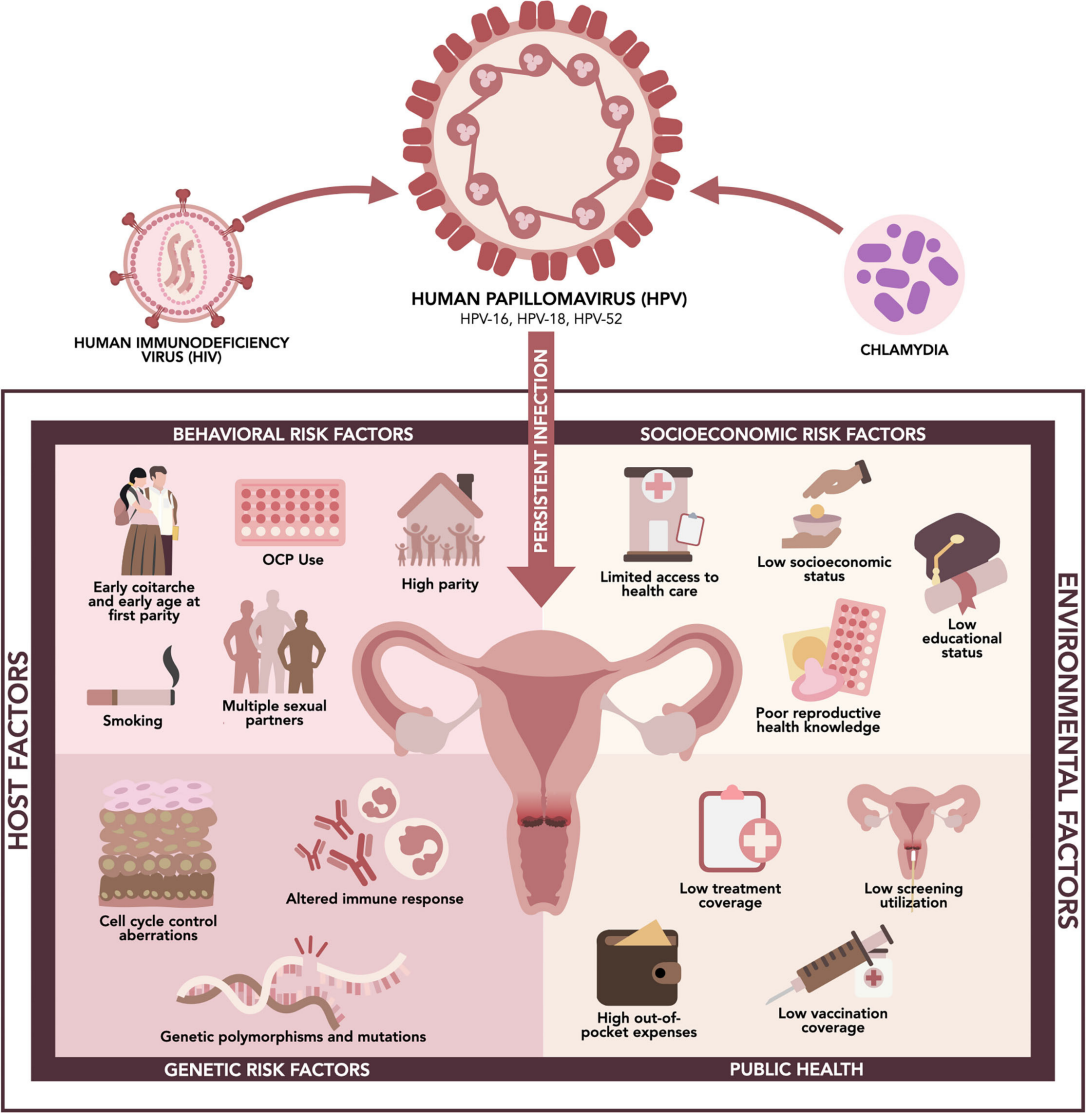
Risk factors associated with high-risk HPV infection and cervical cancer in the Philippines.

Cervical cancer is the second leading cancer among women in the Philippines despite being the fourth leading cancer among women globally, next to breast, colorectum, and lung cancers (30). This may be due to a higher prevalence of risk factors, which are related to increased exposure to HPV or decreased immunologic ability to clear the virus, or a lack of access to essential health services in the country (31). There are no extensive, consolidated studies investigating the burden of risk factors for cervical cancer in the Philippines. However, it is said that risk factors in the Philippines are like those reported in other countries (32). We summarized the potential host and environmental risk factors that contribute to persistent HPV infection and cervical carcinogenesis in Filipino women (Figure 1).

### Microbial Risk Factors

Human immunodeficiency virus (HIV) infection is a significant risk factor for HPV infection, persistence, and associated cancers. This is related to suppression of the immune system, which usually clears HPV in most infected women, and increases the risk of exposure among sexually active adults (33). A meta-analysis showed that an estimated 33,000 new cases of cervical cancer, corresponding to 5.8% of new cases, occurred among women with HIV—a six-fold higher risk than those without HIV. This was lower in the Southeast Asia region, with 1.4% of new cases in people living with HIV (PLHIV) (34). However, this is of paramount concern in the Philippines since the country faces the fastest growing HIV epidemic in the Western Pacific region, increasing from 1 case per day in 2008 to 28 cases per day in 2022. In 2020, around 5% of the 16,700 new cases were females. There were around 115,100 PLHIV in the Philippines in 2020, which is estimated to rise to 331,500 by 2030 (35, 36).

Chlamydia infection caused by *Chlamydia trachomatis* is also an essential co-factor for cervical carcinogenesis. It can cause chronic inflammation, disrupt epithelial integrity, and induce cervical metaplasia. These effects may lead to HPV viral load enhancing, genome integration, and genomic instability, synergizing cervical cancer transformation (37). A hospital-based case-control study from 7 countries, including the Philippines, showed that the odds of squamous cell invasive cervical carcinoma was higher in *C. trachomatis* seropositive women (odds ratio (OR) 1.80; 95% confidence interval (CI) 1.22–2.66), which increased with higher *C. trachomatis* titers in women under 55 years of age (38). Another case-control study conducted on Filipino women showed no significant difference in the prevalence of other sexually transmitted infections (*Ureaplasma* spp., *Mycoplasma* spp., and *C. trachomatis*) between cervical cancer and control group (chronic cervicitis patients). However, this study showed that 22.73% of HPV-positive patients were co-infected with *Ureaplasma* spp. and 9.09% with *Mycoplasma* spp. (16).

### Behavioral Risk Factors

HPV infection is a sexually transmitted infection, hence sexual behavior determines exposure to the virus (39). In the Philippines, around 12% of 15–19 years old were sexually active, increasing to 40% among 20–24 years old, and 47% among 25–27 years old (40). The median age at first sexual intercourse among women of reproductive age was 21.2 years in 2017. In this cohort, 18%, 56% and 73% engaged in sexual intercourse before the age of 18, 22 and 25, respectively (41). In addition, an interval of fewer than 3 years between menarche and coitarche may represent a critical time window for establishing persistent infection and the development of precancerous lesions. In one study, women who had their first sexual intercourse within 3 years of menarche had greater odds of cytologic abnormalities (OR 1.65; 95% CI 1.02–2.68) and CIN 2/3 or adenocarcinoma *in situ* (OR 3.56; 95% CI 1.02–12.47) (42). Proposed mechanisms for this include decreased cervical mucus from decreased progesterone during anovulatory cycles in the initial years following menarche, and elevated estrogen after puberty, which is accompanied by rapid changes in the squamo-columnar junction that increase susceptibility to sexually transmitted infections. The immature cervix may also have increased areas of metaplastic epithelium, thus posing greater vulnerability to HPV infection and neoplastic changes (42, 43).

Early age at first sexual intercourse is associated with an increased risk of HPV infection due to increased exposure and riskier sexual behavior such as having unprotected sex and having multiple sexual partners (39). A meta-analysis including 41 studies showed that the number of sexual partners was associated with the occurrence of non-malignant cervical disease (OR 1.82; 95% CI 1.63–2.00) and invasive cervical carcinoma (OR 1.77; 95% CI 1.50–2.05) (44). In the Philippines, around 34% of sexually active young Filipinos have multiple sexual partners, representing around 1.6 million in the 15–27 years cohort (40).

Age at first parity is a significant risk factor in cervical cancer (45). One possible mechanism being proposed is that during the first pregnancy, the transformation zone gains atypical features with neoplastic potential due to increased size and increased amounts of metaplastic epithelium, similar to changes and vulnerabilities of hormones and the cervix during menarche (42, 43). In a pooled analysis of case-control studies from eight developing countries, including the Philippines, there was an increased risk of cervical cancer as the age at first pregnancy decreased, associated with sexual activity. For those who had an age of sexual debut and first pregnancy on or before 16 years old, the OR was 2.36 (95% CI 1.82–3.07), while for those with coitarche and first pregnancy at 17–20 years old, the OR was 1.93 (95% CI 1.58–2.36) (39). This is of significant demographic repercussions in the Philippines as 15% of women aged 25–49 years were married by age 18, and 1 in 3 women were married by age 20. Childbearing came within 1 year of marriage, and 7% of women had their first birth by age 18 (41).

High parity is also considered a significant risk factor for cervical cancer, as shown in a multi-center study from eight case-control studies showing a direct association of high parity with HPV infection. The odds ratio for seven full-term pregnancies or more was at 3.82 (95% CI 2.66–5.48) as compared to nulliparous women, and 2.25 (95% CI 1.57–3.22) as compared to women with one or two pregnancies (45). This was consistent with another study where nulliparity served as a protective factor with a relative risk (RR) of 0.69 (95% CI 0.60–0.78) and 0.94 (95% CI 0.74–1.18) for squamous cell and adenocarcinoma, respectively, as compared to the RR of 1.50 (95% CI 1.43–1.59) and 1.36 (95% CI 1.22–1.52) for those with 3–4 full-term pregnancies, and 2.08 (95% CI 1.95–2.23) and 1.61 (95% CI 1.37–1.90) for those with ≥5 full-term pregnancies (46).

The use of oral contraceptive pills (OCP) is associated with an increased risk of invasive cervical cancer. This risk increases with increasing duration of use and declines after cessation of OCP use. In less developed countries, the use of OCP for 10 years from around age 20–30 was estimated to increase the cumulative incidence of invasive cervical cancer from 7.3 cases per 1,000 women to 8.3 cases per 1,000 women by age 50 (31). A multi-center hospital-based case-control study, including the Philippines, reported an OR of 2.82 (95% CI 1.46–5.42) with 5–9 years of OCP use, increasing to 4.03 (95% CI 2.09–7.79) with 10 years of use or longer (47). A possible explanation is that sex steroids such as estrogen and progesterone may bind to transcriptional regulatory regions on the HPV DNA, which causes an increase in transcription of oncogenes. Since these hormones also interact with hormone receptors in cervical tissue, they may enhance the expression of E6 and E7 oncogenes of HPV (48, 49).

Smoking is a well-established risk factor for cervical cancer, as supported by several studies (31, 50–53). A pooled analysis of 10,577 women showed that current tobacco smoking is associated with a significant risk of HPV infection with an increasing OR as the number of cigarettes smoked per day increases compared with never-smokers. For <5 cigarettes, 5–14 cigarettes, and >15 cigarettes per day, the corresponding ORs were 1.21 (95% CI 0.95–1.54), 1.39 (95% CI 1.04–1.87), and 2.01 (95% CI 1.32–3.08), respectively (52). Another study showed higher HPV types 16 and 18 DNA load in current smokers (53). Possible mechanisms include direct exposure of DNA in cervical epithelial cells to components of cigarettes and abnormalities in the immune system of smokers, which may cause a substantial decrease in the number of Langerhans cells in the cervices of smokers (54). In the Philippines, around 5% of women of reproductive age smoke a tobacco product, of which 41% smoke <5 cigarettes daily, 23% smoke 5–9 cigarettes, 24% smoke 10–14 cigarettes, and 12% smoke more than 15 cigarettes. However, many women have secondhand smoke exposure: 28% were exposed to secondhand smoke inside the home daily, and an additional 8% were exposed on a weekly basis (41).

### Genetic Risk Factors

Although the molecular mechanisms of HPV-associated cancer development are not well established, there have been several immune response genes from host innate immunity and adaptive immune response associated with regression, persistence, or progression of HPV. Since most individuals can eliminate the virus in 12–24 months without intervention, the role of host genetic differences will be deemed necessary in determining the risk of developing cancer. Polymorphisms and variations in these genes may confer susceptibility or prediction to the development of cervical cancer. However, these findings need further evaluation (55). To our knowledge, there was only one study from the Philippines that investigated these genetic factors (56).

Genes that interact with HPV E6/E7 oncoproteins have been investigated with cervical cancer development (55). Of particular importance is the role of mutations in the tumor suppressor gene *TP53* on HPV-related cancers. *TP53* codes for p53, a nuclear transcription factor which activates target genes that facilitate cell cycle arrest, allowing cells to either repair damaged DNA or undergo apoptosis in the presence of irreparable DNA damage (57, 58). It plays a key role in almost all cancers by regulating and maintaining genomic integrity, and it is reported that over 50% of human cancers carry loss-of-function mutation in p53 (57). The ability of E6 oncoprotein to cause degradation of p53 is important in the survival of HPV-positive neoplastic cells. Indeed, this is a special feature of high-risk HPV types and is seldom associated with malignant lesions in low-risk HPV genotypes (59). A meta-analysis by Tornesello et al. (59) which included 1,353 cervical tumors found that non-synonymous mutations in the DNA-binding domain of *TP53* were found in 13.3% for adenocarcinoma and 5.9% in squamous cell carcinoma (59). The mutations in codons 175, 248, and 273 were commonly mutated in both types of cervical cancers. Additionally, the frequency of *TP53* mutations was highest in Asians at 19% compared to those in North America at 4% (59). However, there are no studies from the Philippines that investigate the frequency of *TP53* mutations in cervical tumors. Aside from *TP53* gene, polymorphisms in breast cancer susceptibility gene 1 (*BRCA1*), BRCA1-associated ring domain protein 1 (*BARD1*) gene, primary microRNA-218 (*pri-miR-218*), and laminin-5 β3 (*LAMB3*) were also associated with increased risk of cervical cancer (55, 60–62).

Pro-inflammatory cytokines, including interleukin 1-beta (IL-1β), tumor necrosis factor-alpha (TNF-α), and interleukin 12 (IL-12), have been implicated in cervical carcinogenesis (55, 63–67). Interestingly, IL-10, an anti-inflammatory cytokine, and B-cell proliferation factors have also been associated with the risk of cervical cancers (55, 68, 69). Polymorphisms in human leukocyte antigen (HLA) genes have also been associated with cervical cancer risk (70). HLA is critical in presenting viral antigens and in inducing of adaptive immune response (70, 71). A variant allele of *IFNG* gene coding for interferon-gamma (IFN-γ), which is essential in defense against viruses and intracellular pathogens and induction of immune-mediated inflammatory responses, has also been associated with increased cervical cancer risk (55, 72). Studies have also identified polymorphisms of *CTLA4* gene associated with increased cervical cancer risk. Cytotoxic T-lymphocyte associated protein 4 encoded by *CTLA4* gene functions as an immune checkpoint and downregulates immune responses (73, 74).

Previous reports showed the association of several gene mutations and polymorphisms with poor prognosis of cervical cancer. *PIK3CA* mutation is associated with poor treatment response and low survival rate, while *MDM2* is associated with the development of cervical cancer and poor prognosis (59, 75). A previous case-control study in the Philippines showed that the *PIK3CA* gene was found mutated in 10.71 % of cervical cancer patients. Around 28.57 % of HPV-negative cervical cancer patients were positive for *PIK3CA* mutation, and 4.76 % tested positive for this mutation among the HPV-positive cervical cancer patients. *MDM2* SNP309 analysis revealed that TG genotype (*p* = 0.03; OR 0.18; 95% CI 0.04–0.76) was associated with lower cervical cancer rates than TT genotype (56).

### Sociodemographic Risk Factors

Often, socioeconomic factors (e.g., income, education, and occupation) are overlooked health factors. However, the most fundamental causes of health disparity can be attributed to socioeconomic inequalities (76). Socioeconomic status underlies three significant determinants of health: access to, use of, and quality of health care; environmental exposure, and health behavior (77).

Poor accessibility of health care is reflected in the high mortality of patients with cervical cancer. The Philippines has the fourth highest age-standardized mortality rate in Southeast Asia with 7.89 per 100,000 women per year, next to Myanmar and Indonesia (14.4), Timor-Leste (8.76), and Cambodia (8.33) (8). This rate is higher than the global age-standardized mortality rate of 7.3 per 100,000 women (30). The overall 5-year survival rate for cervical cancer in the Philippines was reported to be 44%. The low survival rate was attributed to cancer being diagnosed at later stages, in addition to treatment being unavailable, inaccessible, or non-affordable (32, 78). This may be reflective of the poverty incidence in the Philippines estimated at 23.7% or 26.14 million Filipinos who live below the poverty threshold of around PHP 12,082 (USD 232) on average for a family of five per month, as of the first semester of 2021(79). Furthermore, with out-of-pocket expenditure comprising 44.7% of health financing in the country as of 2020, any health-related concerns may lead to financial catastrophe and impoverishment, thus serving as a massive barrier to health in many Filipinos (76, 80).

Related to health care access is health-seeking behavior and health literacy, as they are also intimately linked to socioeconomic factors. Health literacy is described as the ability to access, understand, appraise, and apply health information in terms of health care, disease prevention, and health promotion (81). According to the maiden National Health Literacy Survey, 51.5% have limited health literacy, reflecting the inadequate and poor distribution of health care services and high cost of healthcare in the country (81). It was also shown that the proportion of people with limited health literacy increased with decreasing levels of educational attainment. This is further seen in a community cross-sectional study on the knowledge, perceptions, and screening behavior on cervical cancer in rural health centers in the Philippines, which showed that only 13.9% of participants had ever had cervical cancer screening (82). The majority of those who had never had cervical screening were higher among those with no formal or primary education, currently unemployed, and household monthly income of less than PHP 5,000 (USD 96). Furthermore, the most common reasons listed for not having screening are lack of money, no signs or symptoms, misconceptions about the procedures, and have never heard or do not understand the meaning of cervical cancer screening (82). In the country, we note that the estimated cost of screening is PHP 1,000 (USD 19), which often requires two to three hospital visits for a pap smear. This limits the access to screening utilization and as such, it is significantly associated with higher education status and financial capability. Vaccination for HPV is also expensive with an estimated cost of PHP 2,400 (USD 46) per vaccinated person (83). Thus, it comes as no surprise that socioeconomic disparities become barriers to essential preventive measures against HPV infection and cervical cancer in the country.

Finally, the social environment, which is greatly affected by socioeconomic status, largely affects the behavioral risk factors for HPV infection as previously discussed above. The risk of sexually transmitted infections may increase due to lack of access to condoms and health education, and early pregnancy and marriage are related to education (40). According to WHO, two out of ten young women gave birth before age 20 in the Philippines, and this was increased to four out of 10 among less-educated women. Out-of-school youth, which is primarily concentrated in urban areas, have a higher risk of teenage pregnancy; however, rural women are twice as likely to become pregnant (40).

Overall, there is a lack of local studies that portray how sociodemographic disparities directly contribute to the high burden of cervical cancer in the Philippines. However, their proxy variables, e.g., low income and educational attainment, high poverty incidence, and poor health literacy, may reflect how women of lower socioeconomic status in the country are rendered more vulnerable to cervical cancer.

## Prevention Strategies to Address HPV Infection and Cervical Cancer in the Philippines

### Current Cervical Cancer Screening Strategies and Challenges in the Philippines

The 5-year survival rate for cervical cancer is 44% due to most cervical cancers being diagnosed in an advanced stage, remaining unchanged between 1980 and 2010; thus, it is crucial to screen for cervical cancer and intervene before the onset of symptoms (83). Cervical cancer screening aims to identify and remove high-grade cervical intraepithelial neoplasia that may be precursor lesions to cancer (84). Currently, there are three methods employed to screen women: cervical cytology, primary human papillomavirus testing, and co-testing. Cervical cytology assesses pathologic changes in cells obtained from the cervix under a microscope (84). Cervical sample obtained for cytology can be fixed directly onto a glass slide (i.e., conventional cytology), or suspended in a transport medium (i.e., liquid-based cytology). Although there is no significant difference in specificity and sensitivity between conventional and liquid-based cytology, the latter provides an added benefit of doing HPV testing and/or genotyping, especially when the result is equivocal (85). Pathologic changes along with non-neoplastic findings such as atrophy or reactive cellular changes are reported *via* the Bethesda system (86). Depending on the results and 5-year risk estimate for developing CIN 3+, patients can be managed *via* immediate treatment with excisions such as loop electrosurgical excision procedure, colposcopy, or surveillance of varying intervals (87).

Since virtually all cervical cancer cases are caused by high-risk HPV, primary HPV testing alone is gaining traction as an acceptable screening method given that HPV testing is more sensitive than Pap smear test, with a small decrease in positive predictive value (88, 89). Currently, there are two primary HPV tests approved by the United States Food and Drugs Administration: Roche Cobas HPV approved in 2014, and BD Onclarity HPV approved in 2018. Cobas HPV assay allows specific identification of HPV types 16 and 18, and pooled detection of HPV types 31, 33, 35, 39, 45, 51, 52, 56, 58, 59, 66, and 68 (90). On the other hand, BD Onclarity HPV assay allows individual identification of HPV types 16, 18, 31, 45, 51, and 52, and concurrent detection of other high-risk HPV types into three groups: 33/58, 35/39/68, or 56/59/66 (91). Lastly, co-testing is a method that combines both cervical cytology and HPV testing. Although co-testing identifies the same number of CIN3+ lesions as HPV testing alone, co-testing has worse specificity and positive predictive value, which in turn requires more colposcopies, hence providing evidence that primary HPV testing may suffice as a cervical screening method (92).

Table 1 shows the current screening guidelines for average-risk women. The latest Clinical Practice Guidelines for the Obstetrician-Gynecologist by Society of Gynecologic Oncologists of the Philippines (SGOP) still includes the 2012 guidelines by Philippine Society for Cervical Pathology and Colposcopy (PSCPC), which contrasts it with the 2012 guidelines by American Cancer Society (ACS), American Society for Colposcopy and Cervical Pathology (ASCCP) and American Society for Clinical Pathology (ASCP) (93, 94). The most updated guidelines were released by the American Cancer Society in 2020, with the following significant changes: (1) primary HPV testing as the preferred screening method, and (2) age of first screening changed from 21 to 25 years due to low cervical cancer incidence and mortality, high incidence of transient infection with HPV, higher risk of adverse obstetric outcomes should a lesion be treated, and favorable benefit-to-harm balance as a result of delaying the age of first screening (95).

**Table 1:** Latest cervical cancer screening guidelines for average-risk women.

**Population**	**2012 Guidelines PSCPC (89)**	**2012 Guidelines ACS-ASCCP-ASCP (90)**	**2020 Guidelines ACS (91)**
<21	No screening	No screening	No screening
21–24	Conventional cytology every year OR Liquid-based cytology every 2 years	Cytology alone every 3 years	No screening
25–29	Conventional cytology every year OR Liquid-based cytology every 2 years	Cytology alone every 3 years	Primary HPV testing every 5 years (preferred) OR Co-testing every 5 years OR Cytology every 3 years
30–65	Conventional cytology every year OR Liquid-based cytology every 2 years	Co-testing every 5 years OR Cytology alone every 3 years	Primary HPV testing every 5 years (preferred) OR Co-testing every 5 years OR Cytology every 3 years
Above 65	Conventional cytology every year OR Liquid-based cytology every 2 years OR Co-testing every 5 years Women with a history of CIN2 or worse should continue screening for at least 20 years	No screening if with: (1) 3 consecutive negative prior screening results. (2) 2 consecutive negative co-testing results within the past 10 years. The most recent test should be within past 5 years. Women with a history of CIN2 or worse should continue screening for at least 20 years	No screening if with: (1) no history of CIN2+ within the past 25 years (2) documented adequate negative prior screening in the past 10 years, which may be either: a) 2 consecutive negative HPV tests b) 2 consecutive negative co-tests c) 3 consecutive negative cytology tests Continue screening as previously described until cessation criteria are met in individuals without conditions limiting life expectancy without sufficient documentation of prior screening
After hysterectomy	No screening if without history of CIN2+ or cervical cancer in the past 20 years	No screening if without history of CIN2+ or cervical cancer in the past 20 years	No screening if without history of CIN2+ in the past 25 years or cervical cancer ever

Visual inspection with acetic acid (VIA) is an acceptable alternative approach to pap smear in low-resource settings. It is a simple test with locally available supplies and can be performed by trained health workers. The result is immediate, appearing as acetowhitening, which allows VIA to be combined with treatment procedures for early cervical lesions such as cryotherapy (96, 97). Numerous studies have been conducted in the Philippines establishing VIA as more sensitive but less specific than pap smear in detecting precancerous lesions (98, 99). In addition to its utility, this screening approach is favored by developing countries because shifting from pap smear to VIA would result in lower healthcare costs with higher health benefits (100). Thus, given its validity and cost-effectiveness, VIA was adopted to be the initial screening approach in the Philippine setting in 2005, with colposcopy with a pap smear or biopsy as confirmatory test following positive VIA. Indeed, in a cost-utility analysis which looked at epidemiologic, cost, and clinical parameters specific to the Philippines from a health systems perspective, high VIA coverage targeting women aged 35–45 years at five-year intervals was found to be the most efficient and most cost-saving screening strategy, reducing cervical cancer cases and deaths by 25% (83).

Despite the introduction of pap smear in the Philippines during the 1990s and subsequent promotion of VIA as an alternative screening approach to a pap smear in 2005, screening utilization remains low. In a household health survey done in 2003, the estimated coverage of cervical cancer screening in women aged 18–69 was 7.7%, with 9.3% coverage in urban areas and 5% coverage in rural areas (101). More recent data showed better yet dismal coverage. In a cohort of women aged 25–55 years residing in an urban area, only 36.8% had undergone pap smears at least once, despite 82.9% being sexually active (102). Among women aged 26–35 years consulting at a private hospital in greater Manila area and a public hospital in southern Philippines, only 48% of the women had undergone pap smear at least once, with even fewer women (31%) undergoing routine pap smear tests (103). Utilization of cervical cancer screening services is even lower in rural areas: a community-based found that only 13.9% of participants had ever had cervical cancer screening, despite 93.8% having heard of screening (82). A health systems survey showed that the average annual coverage of cervical cancer screening using VIA in the urban group consisting of major cities in Metro Manila was 5.50%, while the coverage in the rural group consisting of municipalities in Albay province southeast of Manila was 0.39% (104).

Among the factors identified that can be attributed to the failure of cervical cancer screening were (1) lack of knowledge about symptomatology of cervical cancer, (2) perception that cancer is fatal and lack of awareness that cervical cancer is treatable, (3) unavailable screening and treatment facilities and expertise, and (4) inconsistent patient adherence to follow-up consults and treatment (105). There are also misconceptions regarding cervical screening. In a study which included 400 women aged 26–35 years of age consulting outpatient a private hospital in greater Manila area and a public hospital in southern Philippines, more women believed that pap smear is done for detecting lower genital infections (75%) than pap smear being done for detecting cervical changes that may lead to cancer (57%) (102). Although 64% agreed that sexually active women should undergo a pap smear test, 52% thought it should only be done when women present with symptoms. Only 67% believed that pap smear tests should be done annually. There was also a discrepancy between attitude and practices regarding pap smear: despite the majority agreeing that women should undergo pap smear tests, only 48% had pap smear tests at least once, and 31% had routine testing (102).

Given the burden of disease in the Philippines, the national government, through the Department of Health and local government units, has started advocating for cervical cancer screening starting in 1999 (32, 106). Advocacy to raise awareness regarding cervical cancer prevention was initiated in 2003 *via* Proclamation No. 368, which declares May as Cervical Cancer Awareness Month (107). As part of the Philippine Cancer Control Program, an organized nationwide Cervical Cancer Screening Program was established in 2005 *via* Department of Health Administrative Order 2005–2006, which included public information and health education, sustainable capacity building, and training and professional education of health workers on case-finding with VIA, and diagnosis with the use of pap smear and colposcopy (106). The national recommendation is to target screening women aged 25–55 years *via* VIA every 5–7 years. In 2020, as part of the effort to ensure equitable access to primary care services, the state-owned Philippine Health Insurance Corporation (PhilHealth), which is responsible for implementing universal health coverage, started coverage of basic outpatient services including pap smear (108). However, an assessment of health facilities in Metro Manila and Albay province showed a disparity in the implementation of cervical cancer screening between urban and rural areas (104). Although most health facilities in both urban and rural groups are oriented to Cervical Cancer Screening Program, only 31.25% of the facilities in the rural group had a screening protocol, compared to 100% of urban facilities. All facilities in the urban group provided screening services, while only 4 out of 16 facilities in the rural group were screening providers. Despite facilities in both urban and rural groups having available instruments for cervical screening, the trained health personnel-to-population ratio in the rural group was 1:1,751, compared to 1:699 in the urban group. As a result of the decentralization of the health system, financial support for the screening program was reliant on the local health budget. Majority of the facilities in rural areas had no budget item for screening instead of relying on the general budget for health (104).

Various strategies were done in selected places to improve the utilization rate, but these are yet to be implemented nationwide. Opportunistic screening programs where cervical cancer screening is offered to patients in the waiting area during regular health consultation services can be utilized to encourage women. In a study done in a public tertiary hospital, implementation of a month-long opportunistic screening program increased the utilization rate of cervical screening from 2 to 27%, with most women availing of screening being married, with high school education, multiparous, had no previous screening, and was not knowledgeable about cervical cancer (109). Health education is also important: in a cohort of female secondary school teachers from a rural area, providing a lecture increased acceptance of VIA from 0 to 78%, with 71.4% of the participants submitting themselves to free VIA testing following the lecture (110). In a large-scale setting, however, a public health education program that is geared toward identifying factors that facilitate or inhibit consultation and developing a community intervention program to improve screening compliance is essential. Using the Health Decision Model to explain health-seeking behaviors (111), the best predictors of compliance to pap smear screening were found to be civil status, level of education, number of children, family history of cancer, and perceived risk of cancer, while cost was a critical inhibiting factor (112). Using the perceived risk of having cancer as a basis, implementing a health education program increased pap smear consultations. In summary, a focused information education must be coupled with accessible and well-equipped screening centers to ensure the success of any nationwide cervical cancer screening program.

### Cervical Cancer Prevention *via* HPV Vaccination

Currently, three prophylactic HPV vaccines are registered with the Philippine Food and Drug Administration: Cervarix, a bivalent vaccine produced by GlaxoSmithKline that prevents HPV types 16 and 18 (4); Gardasil, a quadrivalent vaccine produced by Merck that prevents HPV types 6, 11, 16, and 18 (3); and Gardasil-9, a nonavalent vaccine produced by Merck that prevents against HPV types 31, 33, 45, 52 and 58 in addition to the coverage of Gardasil (5). All three HPV vaccines are given intramuscularly, with two doses administered at 0 and 6 months to persons aged 9–14 years and three doses administered at 0, 2, and 6 months to persons aged 15 years and older. Cecolin, a bivalent vaccine, which has been licensed in China, is currently undergoing World Health Organization prequalification process (113).

At a population level, HPV vaccination has been found to reduce the prevalence of high-risk HPV types, anogenital warts and high-grade cervical abnormalities, as evidenced by a meta-analysis of 65 studies involving 60 million individuals who followed up for 8 years (114). The prevalence of HPV types 16 and 18 declined by 83% (RR 0.17, 95% CI 0.11–0.25) and 66% (RR 0.34, 95% CI 0.23–0.49) in girls aged 13–19 years and women aged 20–24 years, respectively, while the prevalence of HPV types 31, 33 and 45 also decreased by 54% (RR 0.46, 95% CI 0.33–0.66) in girls aged 13–19 years. HPV vaccination was also a protective factor for two clinical outcomes: (1) anogenital warts, with RRs of 0.33 (95% CI 0.24–0.46) and 0.46 (95% CI 0.36–0.60) in girls aged 13–19 years and women aged 20–24 years, respectively; and (2) CIN 2+ with RRs of 0.49 (95% CI 0.42–0.58) and 0.69 (95% CI 0.57–0.84) in girls aged 13–19 years and women aged 20–24 years, respectively (114).

Prior to the widespread introduction of HPV vaccines in the Philippines, the acceptability of these vaccines was determined in various studies. In an exploratory study involving 195 women consulting at charity clinics in a tertiary hospital with daughters aged 12–15 years, HPV vaccination was acceptable to 75.4% of women despite only 56.4% identifying HPV as a sexually transmitted infection and 31.8% associating HPV with cervical cancer (115). In a cohort of female adolescents aged 14–19 years consulting at a pediatric specialty hospital, 53% heard about the HPV vaccine, and the majority were willing to get vaccinated if given free (116). A study involving commercial sex workers (CSWs) in Angeles City, Pampanga province, reported that despite 87% having poor practices on cervical cancer prevention, which were attributed to inadequate knowledge and poor health-seeking behavior, the majority of the CSWs have favorable attitudes regarding HPV vaccination (117). The principal reason for the non-acceptance of HPV vaccines was the perceived high cost of vaccination. A community-based study involving 435 adult women reported that HPV vaccine acceptance was contingent on affordable pricing, with 54% accepting at a low price and only 30% and 31% accepting at a moderate and high price (118). Vaccine acceptance was lower in men, with 22–39% of men aged 18–31 years old accepting of HPV vaccination. However, it remained contingent on affordable pricing (117). Other reasons included young age, painful injection and sexual inexperience for pediatric patients aged 10–19 years, and concern that HPV vaccination could promote unsafe sexual behaviors (115).

In 2015, the national HPV immunization program was partially introduced in the Philippines with either bivalent or quadrivalent vaccine through Department of Health Memorandum No. 2015-0316 (9). Initially a community-based immunization program, the government changed the protocol to a school-based immunization program targeting young girls aged 9–14 years to ensure high coverage and minimal dropout rate. Despite being free, which was the predominant factor affecting vaccine acceptance (115, 118, 119), the Philippines still ranked last among low- to middle-income countries on HPV program coverage (7). As of 2020, 23% of the female target population received the first dose of the HPV vaccine, virtually unchanged from 2019, while 5% received the last dose, up from 3% (11). Due to the coronavirus disease 2019 (COVID-19) pandemic, which resulted in suspension of in-person classes for most of 2020 and all of 2021, strategies to continuously provide immunization service such as having stations at permanent health facilities or temporary posts at multi-purpose town halls, and door-to-door approach are being employed (120). Once in-person classes resume, the program will be reverted to school-based immunization.

There are limited studies assessing the implementation of the national HPV immunization program. A cost-effectiveness analysis done in 2017, which has yet to be taken into consideration by the implementing health agency, found that the 2-dose bivalent HPV vaccine administration to 13-year-old Filipino girls prevented additional 986 cervical cancer cases and 399 deaths from cervical cancer, with 555 additional quality-adjusted life-year compared to the 2-dose quadrivalent vaccine (121). It also would result in lower health costs, saving PHP 228.1 million (approximately USD 4.4 million). Studies assessing health systems and socio-cultural determinants affecting intent to vaccinate are needed to understand gaps in implementation resulting in low HPV vaccination coverage better.

## Treatment of Persistent HPV Infection and Cervical Cancer in the Philippines

A persistent HPV infection is defined as having positive HPV tests at two consecutive time points: at baseline and on follow-up. The minimum duration to classify a woman as having persistent HPV infection varies across studies, with 30% using <6 months as minimum duration, 45% using 6–12 months, and 25% using more than 12 months (122). Type-specific HPV persistence (testing positive for the same HPV type), especially with high-risk HPV, is associated with treatment failure resulting in incomplete removal of HPV infection, recurrent cervical intraepithelial neoplasia (CIN), or progression to cancer. This should be differentiated from an incident HPV infection, a re-infection with a new HPV type not associated with the primary cervical lesion. Routine HPV testing after treatment of CIN 2/3 is recommended for early detection of disease recurrence or progression since these high-grade lesions are associated with 60–80% of persistence (123, 124).

A systematic review of 45 studies involving over 6,000 women showed a decline in median post-treatment HPV persistence with increasing follow-up time (123). Reported post-treatment HPV persistence estimates varied depending on treatment type, patient age, HPV type grouping, HPV detection method, and minimum interval between the two testing points to define HPV persistence. CIN treatment successfully removed HPV from the cervical tissue, but this does not preclude cases where HPV is still present in the vaginal mucosa and may cause re-infection of the cervix during follow-up. None of the included studies evaluated the vaginal/vulvar HPV prevalence after treatment. Overall, when considering the type of treatment, conization and loop electrosurgical excision procedure (LEEP) were able to clear HPV infection within 12 months of procedure better than cryotherapy (123).

Another review included 86 studies providing data on over 100,000 women (125). The investigators found that persistence varied across studies but was primarily mediated by study region and HPV type. HPV types 16, 31, 33, and 52 were the most persistent genotypes with a weighted median duration of HPV detection of high-risk HPV (9.8 months), persisting longer than low-risk HPV (8.4 months). HPV type 16 persisted the longest at 12.4 months and is reported as the most critical risk factor for recurrence. HPV-positive women with normal cytology had a median duration of 11.5 months for any HPV type in general, and 10.9 months for high-risk types (125). Since half of HPV persistent infections persist past 6–12 months, repeat HPV testing at 12-month intervals would be able to identify women at increased risk for CIN 2/3 due to these persistent infections.

Several studies could not find sufficient evidence from randomized controlled trials to provide the best post-treatment surveillance strategy (126, 127). While high-risk HPV testing is more sensitive than follow-up cytology for detecting post-treatment high-grade lesions; there is currently no consensus on how this may be best applied. Studies on algorithms for post-treatment HPV testing should consider the testing interval, follow-up time, number of post-treatment tests, and assays used.

Treatment of HPV persistent infections according to the American Society of Colposcopy and Cervical Pathology (ASCCP) guidelines emphasize a risk-based strategy aligned with current knowledge on HPV natural history and cervical carcinogenesis (87). More frequent surveillance, colposcopy, and treatment are recommended for high-risk patients, while low-risk patients may have a deferral of colposcopy and follow-up at longer surveillance intervals with a return to routine screening. The type of HPV and duration of infection will determine the patient's risk with CIN 3. Current results combined with history and the immediate CIN 3 risk for each patient are examined. A risk >4.0% will require colposcopy or treatment (87). Excisional treatment such as LEEP, cold knife conization and laser cone biopsy is preferred over ablation treatment such as cryotherapy, laser ablation and thermoablation. This risk-based strategy is a challenge to implement in the Philippines as cytology-based testing remains the most frequent screening modality, and actual figures for HPV-based testing based on a broader sample population that is more representative of the current Philippine situation have not been reported (8).

In their clinical practice guidelines, the Society of Gynecologic Oncologists of the Philippines (SGOP) advises post-treatment monitoring after 6 months then annually for 3 years. They further support the use of vaccination against HPV types 16 and 18 as efficacious against persistent HPV infection and CIN 2/3 (93, 128, 129). Due to the ongoing COVID-19 pandemic, local consensus statements on cytology screening and colposcopy have reduced testing to only cases where immediate action is necessary, making it difficult to perform studies that will further elucidate factors involving HPV persistence in the population (130).

Evidence supports post-treatment or therapeutic vaccination to reduce the risk of clinical disease relapse after treatment (131). A study by Ferris et al. further suggests that women older than the age typically targeted by HPV vaccination programs are at risk for incident and incident-persistent HPV anogenital infections, depending on sexual behavior. The latter calls for a possible recalibration of algorithms to include older women for primary and secondary (post-treatment) vaccination (132). Other modes of treatment for persistence still under investigation include antivirals (i.e., cidofovir) and immunomodulators (i.e., imiquimod) (133).

Moreover, newer studies have also uncovered the beneficial effect of the cervicovaginal microbiome, precisely certain *Lactobacillus* spp., in inhibiting cellular cervical pathogenesis by producing bacteriocins, lactic acid, and hydrogen peroxide. Ongoing studies may soon further elucidate the underlying physiology of microbe- and host-microbe interactions with HPV infection (134, 135).

Cervical cancer is diagnosed *via* histopathologic examination of tissue obtained from suspicious-looking cervical mass *via* biopsy (136). Since 2018, imaging studies such as radiography, ultrasound, computed tomography, magnetic resonance imaging may be used in addition to clinical examination in assigning cancer stage. Depending on the stage, cervical cancer is managed *via* surgery, radiotherapy, chemotherapy, or a combination of the three modalities. Specifics of clinical management of cervical cancer were comprehensively discussed in 2018 International Federation of Gynecology and Obstetrics (FIGO) Cancer Report, with an update published in 2021 (136, 137).

In the Philippines, only an estimated 50–60% of cervical cancer patients receive some form of treatment (138). Given that 23.7% of Filipinos live below the poverty threshold, and that 44.7% of health financing in the country comes from out-of-pocket expenses, seeking treatment may be economically catastrophic to many Filipinos (76, 79, 80). Recognizing the massive financial risk, various financial risk protection mechanisms were set in place by numerous government agencies over the years. PhilHealth covers cancer treatment as part of its Z Benefit Packages for Cancer (139). Medicines for gynecologic cancers including cervical cancer not covered by PhilHealth will be given for free *via* the Cancer, Supportive Care and Palliative Care Medicines Access Program (CSPMAP). This program is funded by the Cancer Assistance Fund, established under Republic Act 11215 or the National Integrated Cancer Control Act passed in 2019 (140). Indigent and financially incapacitated patients can avail of medical and financial assistance in accordance with Republic Act 11463 (141).

## Conclusion

Although the Philippines has achieved significant milestones in the control and prevention of cancer, such as passing the National Integrated Cancer Control Act (NICCA) in 2019 (142), cervical cancer and other cancers related to HPV infection remain important public health problems in the country. Primary and secondary cervical cancer prevention activities are still implemented locally in the Philippines, with no established national programs on cervical cancer screening and HPV vaccination. Cervical cancer-related services are not widely available in local health centers or units, particularly outside the main urban centers (143). To achieve WHO-specified country targets for cervical cancer and HPV infection by 2030, health policy reforms guided by locally derived research findings should be implemented.

With the information collated and analyzed in this review, we propose the following national research agenda related to cervical cancer and HPV infection in the Philippines. First, implementation research should be done to develop a locally applicable system on how to implement a national program on cervical cancer screening and HPV vaccination. These include highlighting opportunities and addressing challenges in quality of health service delivery, availability of medical supplies including vaccines and screening materials, presence of trained health personnel to administer the services, establishment of a national registry that can provide timely data to implementers and policymakers, and cost-effectiveness of different interventions (144). Second, epidemiological studies should be done to give locally derived data on community-based prevalence, and genotype distribution of HPV infection in women and men since most data in the Philippines are from hospital-based studies. To address this research gap, we are currently conducting a community-based cohort study among Filipino women in rural and urban Philippines to determine the HPV prevalence and genotype distribution and identify factors influencing the acquisition, clearance, and persistence of HPV infection. This study will update the more than 20-year-old data on the population-based prevalence of HPV infection that can be used to strengthen health policies and programs on cervical cancer and HPV infection (83). Third, clinical research on the cost-effectiveness and effectiveness of different traditional and novel treatment strategies should be done to guide the Philippine Health Insurance system on what treatment options should be included in their packages. Lastly, social science studies should also be prioritized to provide data on the perception and acceptability of the different interventions on the target population.

## Author Contributions

RL, OT, and SdP-S edited and proofread the manuscript. All authors read, approved the final manuscript, and drafted the manuscript.

## Funding

RL, LC, GP, OT, IT, and SdP-S are past and current trainees of the MD-Ph.D. in Molecular Medicine Program, supported by Department of Science and Technology—Philippine Council for Health Research and Development (DOST-PCHRD), and administered through University of the Philippines Manila. Funding support for this publication was provided by MD-Ph.D. in Molecular Medicine Program.

## Conflict of Interest

The authors declare that the research was conducted in the absence of any commercial or financial relationships that could be construed as a potential conflict of interest.

## Publisher's Note

All claims expressed in this article are solely those of the authors and do not necessarily represent those of their affiliated organizations, or those of the publisher, the editors and the reviewers. Any product that may be evaluated in this article, or claim that may be made by its manufacturer, is not guaranteed or endorsed by the publisher.
